# Regional and demographic disparities in low-risk hospital deliveries in Brazil’s Public Health System

**DOI:** 10.31744/einstein_journal/2026GS1911

**Published:** 2026-03-18

**Authors:** Marina Martins Siqueira, Gustavo Yano Callado, Lucas Hernandes Corrêa, Gabriely Rangel Pereira, Eduardo Félix Martins Santana

**Affiliations:** 1 Faculdade Israelita de Ciências da Saúde Albert Einstein Hospital Israelita Albert Einstein São Paulo SP Brazil Faculdade Israelita de Ciências da Saúde Albert Einstein, Hospital Israelita Albert Einstein, São Paulo, SP, Brazil.; 2 Centro de Estudos e Promoção de Políticas de Saúde Sociedade Beneficente Israelita Brasileira Albert Einstein São Paulo SP Brazil Centro de Estudos e Promoção de Políticas de Saúde, Sociedade Beneficente Israelita Brasileira Albert Einstein, São Paulo, SP, Brazil.; 3 Department of Obstetrics Universidade Federal de São Paulo, São Paulo SP Brazil Department of Obstetrics, Universidade Federal de São Paulo, São Paulo, SP, Brazil.

**Keywords:** Epidemiology, Delivery, obstetric, Cesarean section, Maternal health, Hospitalization, Healthcare disparities, Pregnancy outcome, Brazil

## Abstract

**Objective:**

To compare the volume, public funding, and in-hospital outcomes of vaginal deliveries and cesarean sections within the Brazilian Public Health System (SUS - *Sistema Único de Saúde* ) across regions and maternal age groups.

**Methods:**

This retrospective, cross-sectional study presents a population-based analysis of low-risk vaginal and cesarean hospital deliveries within the SUS from 2009 to 2023 using publicly available data from the SUS Hospital Information System. Trends in delivery mode distribution were assessed using the chi-square test, whereas in-hospital mortality rates, length-of-stay, and average public financing were compared between the groups using the Mann-Whitney test.

**Results:**

Over the study period, vaginal deliveries decreased by 39%, whereas cesarean sections increased by 3%, leading to a rising proportion of cesarean births within the SUS despite an overall decline in total deliveries in Brazil. The cesarean section rate increased from 32.4% in 2009 to 44.6% in 2023, and regional disparities were observed. Despite the SUS promoting vaginal delivery for low-risk pregnancies, cesarean section rates increased across all states except Roraima. In-hospital mortality rates were numerically higher for cesarean sections, particularly among women aged ≥40 years, although statistical significance was not observed. Cesarean sections were also associated with a longer length-of-stay and higher public funding.

**Conclusion:**

Despite policies favoring vaginal births, rising cesarean section rates in the SUS reveal persistent structural, cultural, and logistical challenges. Higher public funding and longer hospital stays add to the healthcare burden. Strengthening primary care, expanding Normal Birth Centers, and optimizing cesarean section use are crucial for balanced and accessible maternity care across Brazil.

## Highlights

■ Analysis of childbirth methods using national databases is essential for informing maternal–fetal health policies and research.

■ This retrospective cross-sectional study examined national and regional delivery mode trends in the Brazilian Public Health System, comparing pre- and post-COVID-19 periods to address gaps in the literature.

■ Marked regional differences were identified in delivery mode trends and associated outcomes.

## In Brief

The rising cesarean section rates in the Brazilian Public Health System, despite policies promoting vaginal birth, reflect persistent structural, cultural, and logistical challenges. Siqueira et al. emphasized the need to strengthen primary care, expand Normal Birth Centers, and optimize cesarean sections to achieve more balanced and equitable maternity care across Brazil.

## Graphical abstract

**Figure:**
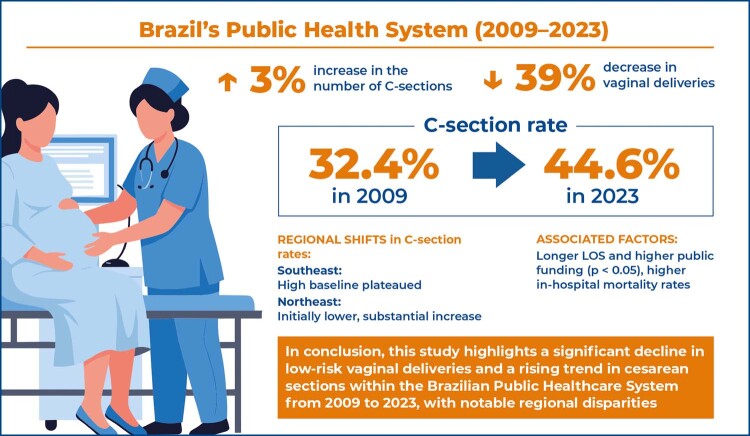

## INTRODUCTION

The analysis of childbirth methods using national databases is pivotal for advancing maternal and fetal health policy and research. These datasets enable the identification of temporal trends and geographic disparities, providing insights into regional and socioeconomic inequalities. For instance, in Friuli Venezia Giulia, Italy, maternal age, breech presentation, and obstetric conditions contributed significantly to higher emergency cesarean rates compared with vaginal delivery rates.^( 1 )^ Such findings underscore the importance of targeted strategies to minimize unnecessary cesarean sections. At the international level, the Euro-Peristat study across 31 European countries revealed substantial variations in obstetric practices, highlighting the need for standardized evidence-based guidelines.^( 2 )^

In Brazil, studying delivery methods is particularly important because of the pronounced regional disparities, socioeconomic influences, and health policy implementation challenges. The country has one of the highest cesarean rates globally, with stark differences between public and private healthcare systems.^( 3 )^ Cesarean rates in private hospitals often exceed 90%, in contrast to lower, though still elevated, rates in the public sector.^( 4 )^ These disparities are strongly influenced by factors such as education level and access to private healthcare insurance. Additionally, Brazil’s high prevalence of cesarean deliveries raises concerns about over-medicalization, which is associated with an increased risk of maternal and neonatal complications.^( 4 )^

Although previous studies^( 5 - 7 )^ have already reported on delivery methods in Brazil, to our knowledge, this is the first study to assess delivery modes over an extended period spanning the pre- and post-COVID-19 pandemic, with a focus on national and regional patterns.

## OBJECTIVE

This study aims to present epidemiological data on delivery modes within Brazil’s Public Health System (SUS - *Sistema Único de Saúde* ), from 2009 to 2023, contributing to a deeper understanding of childbirth practices and their implications.

## METHODS

This retrospective cross-sectional study includes a population-based analysis of vaginal and cesarean hospital deliveries performed within the SUS between 2009 and 2023. The de-identified data were publicly available from the SUS Hospital Information System (SIH - *Sistema de Informações Hospitalares* ). Data were collected throughout December 2024 using the SUS desktop tabulator (TABWIN), and organized using Microsoft Excel spreadsheets.

Vaginal and cesarean deliveries in the public healthcare system were exclusively performed in hospital settings, irrespective of birth risk. To improve comparability of outcomes between delivery types, the analysis was restricted to low-risk hospital deliveries not performed with sequential or combined procedures, such as delivery followed by tubal ligation. Procedures were identified through the SUS Table of Procedures, Medications, Orthoses, Prostheses, and Special Materials^( 8 )^ in which each procedure provided by the SUS has a unique code used in both hospital and outpatient information systems. Vaginal deliveries were tracked using two procedures (codes: 03.10.01.003-9 and 03.10.01.005-5). The first involves assistance in low-risk births with vaginal extraction of the conceptus and cephalic or breech presentation. In the case of stillbirth, a normal birth is considered when the gestational age is greater than 20 weeks, the height of the fetus is equal to or greater than 25cm, or the body weight of the fetus is equal to or greater than 500g. The second also considers assistance in low-risk births with vaginal extraction of the conceptus but performed in Normal Birth Centers (CPNs *- Centros de Parto Normal* ), intended for humanized hospital care for the parturients and newborns. Cesarean deliveries were identified by one procedure (code: 04.11.01.003-4), which was described as a low-risk surgical birth performed through an incision in the uterus.

The results were stratified by the parturients’ age, considering the age groups used in the Brazilian Census and the cutoffs for adolescent pregnancy and advanced maternal age (<15 years, 15-19, 20-24, 25-29, 30-34, 35-39, ≥40 years).

The analyses included the comparison of vaginal and cesarean deliveries regarding procedure volume, population-adjusted rates, SUS funding, in-hospital mortality, and in-hospital length-of-stay (LOS), distributed across the parturients’ age groups (in line with the age groups considered by IBGE Census and SUS information systems) and the Brazilian regions of hospital admission. Funding amounts in Brazilian Reals (BRL) were converted to US dollars (US$) using the exchange rate on December 31, 2016 (US$ 1=BRL 3.2535), corresponding to the median date between the first and last cases evaluated, as adopted in previous epidemiological analysis using SIH.^( 9 , 10 )^

The χ^2^ test was used to evaluate trends in volume distribution by delivery type throughout the study period. In-hospital mortality rates and average costs/funding were compared between the groups using the Mann-Whitney U test. For all tests, the level of statistical significance was set at p<0.05. The collected data did not undergo any transformation for distribution normalization, which is suitable for real-world data analysis.^( 9 , 10 )^

## RESULTS

**Figure 1:**
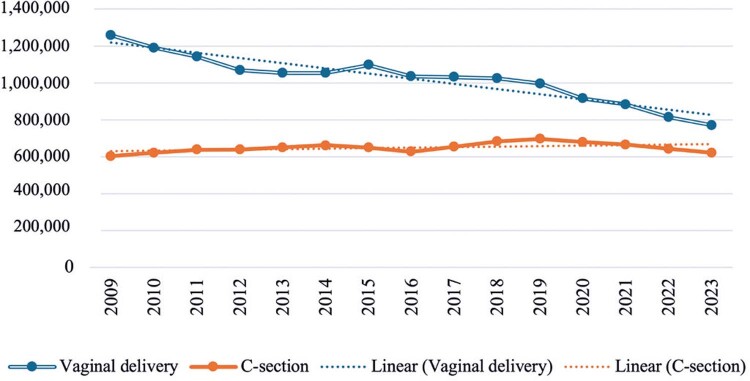
Absolute number of vaginal and cesarean deliveries per year

Source: Developed by the authors with data extracted from SIH/SUS using TABWIN, considering SUS/SIGTAP procedures 03.10.01.003-9, 03.10.01.005-5, and 04.11.010034.

During the study period, a relatively consistent decline in the number of vaginal deliveries was observed in Brazil, from approximately 1.26 million in 2009 to less than 772,000 in 2023, representing a 39% reduction. In contrast, the number of cesarean sections showed a modest increase of 3%, from 603,800 to 622,400. Although vaginal deliveries significantly outnumbered cesarean sections in 2009, this gap progressively narrowed each year ( Figure 1 ). The χ^2^test for temporal trends revealed χ^2^ of 121.335, p<0.001, indicating a statistically significant difference between delivery modes and suggesting a decreasing trend in vaginal deliveries and an increasing trend in cesarean deliveries over the study period ( Table 1S , Supplementary Material).

**Figure 2:**
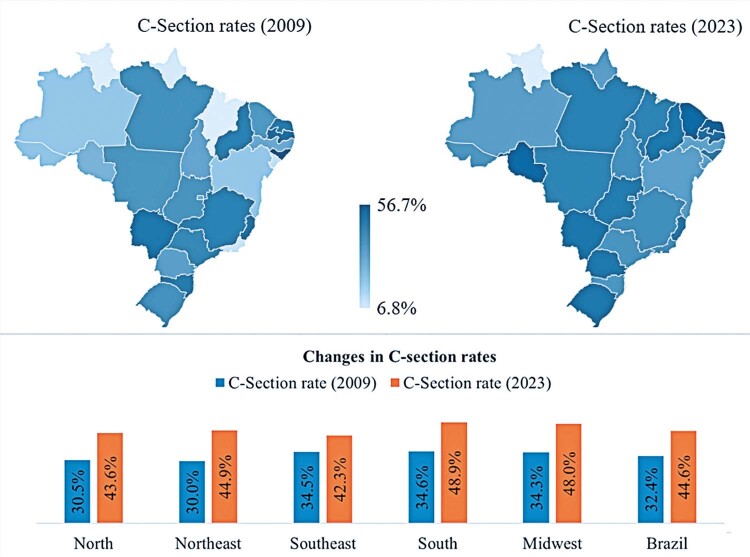
Cesarean section rates by Brazilian region and Federal Units (2009 *versus* 2023)

Notes: The cesarean section rate is computed by dividing cesarean section deliveries by the total number of cesarean and vaginal deliveries in each given year.

Source: Developed by authors, with data extracted from SIH/SUS via TABWIN, considering SUS/SIGTAP procedures 03.10.01.003-9, 03.10.01.005-5, and 04.11.01.003-4.

From 2009 to 2023, Brazil and all its regions experienced an increase in the cesarean section rate, defined as the proportion of cesarean sections relative to total deliveries during the study period ( Figure 2 ). Nationally, the cesarean section rate increased from 32.4% in 2009 to 44.6% in 2023, with the consecutive annual increased throughout the analysis period, except in 2015. The exception was the northern state of Roraima, where the rate declined significantly from 19.4% to 6.8%, the lowest rates observed nationally in those respective years. All other states showed an increase in cesarean section rates, albeit to varying degrees. The smallest increase occurred in the southeast region, Brazil’s most urbanized and economically developed region, which had the highest cesarean rate in 2009 and demonstrated a slower rate of increase over the study period. In contrast, the Northeast, which initially had the lowest regional rate in 2009, showed a substantial increase over the years. The sociodemographic features of the Brazilian regions and the list and map of states per region are depicted in Table 2S and Figure 1S, Supplementary Material .

**Figure 3:**
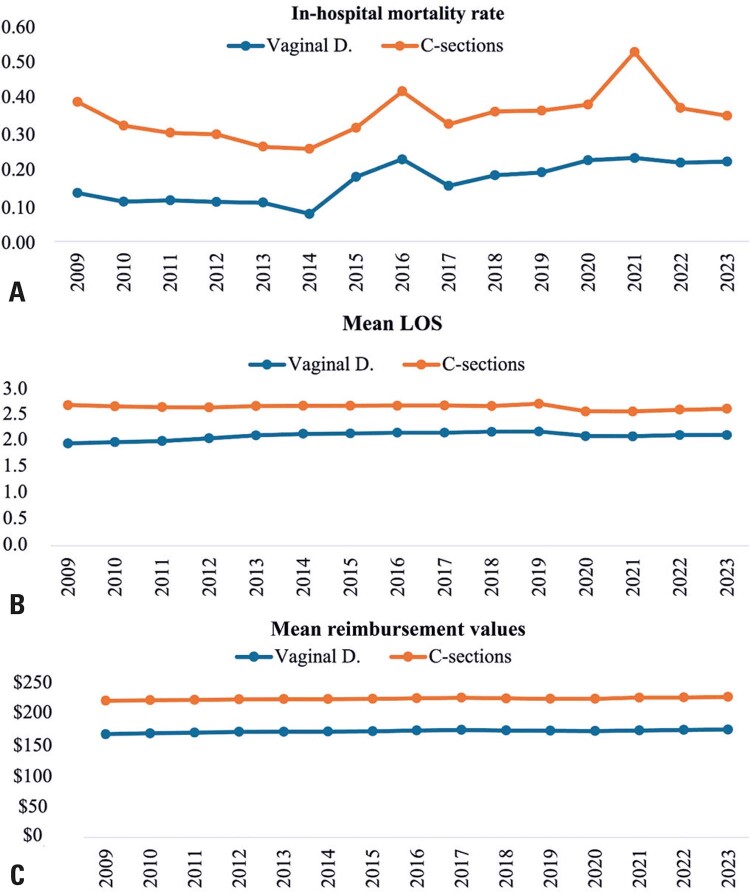
(A) In-hospital maternal mortality rate, (B) Mean length-of-stay - in days, and (C) Average SUS financing values, in US$, by delivery type (2009-2023)

Notes: Mortality rates represent the number of deaths during hospitalizations per 1,000 hospitalizations.

Source: Developed by the authors, with data extracted from SIH/SUS via TABWIN, based on SUS/SIGTAP procedures 03.10.01.003-9, 03.10.01.005-5, and 04.11.01.003-4.

When comparing outcomes between vaginal and cesarean deliveries, cesarean sections consistently exhibited higher in-hospital mortality rates, longer mean in-hospital LOS, and greater average SUS funding amounts across all years analyzed ( Figure 3 ). In terms of in-hospital mortality rates, both vaginal deliveries and cesarean sections showed reductions from 2009 to 2014, followed by an overall increase in the subsequent years. A notable peak in the cesarean section mortality rate was observed in 2021. Mean LOS changed only modestly throughout the study period for both delivery types ( Figure 3 ).

Disaggregating the results by Brazilian region and maternal age groups, higher in-hospital mortality rates, mean LOS, and SUS funding were consistently observed for cesarean sections than for vaginal deliveries. Among both delivery types, particularly within the cesarean section group, women aged 40 years or older demonstrated higher in-hospital mortality rates, longer mean LOS, and greater average SUS financing values (Table 3S, Supplementary Material ).

Regional differences were also noted in 2023, varying according to the delivery type. The northeast region recorded the lowest LOS and average SUS funding across all age groups for both cesarean sections and vaginal deliveries. Conversely, the longest LOS was observed in the southeast for vaginal deliveries and in the north for cesarean sections. Additionally, the southern region had the highest average SUS financing values for both delivery types compared to other regions ( Table 3S , Supplementary Material ).

Statistical differences in outcomes, including average SUS funding, mean LOS, and in-hospital maternal mortality rates, between vaginal deliveries and cesarean sections across Brazilian states were evaluated using the Mann-Whitney U test. A statistically higher mean public financing and mean LOS were observed in the cesarean section group compared with the vaginal delivery group (p<0.05). Although the number of deaths and in-hospital mortality rates were numerically higher for cesarean sections in all Brazilian regions, no statistically significant difference was observed between delivery modes ( Tables 4S , 5S , and 6S , Supplementary Material ).

## DISCUSSION

The SUS guidelines on delivery modes emphasize vaginal delivery as the preferred method for low-risk pregnancies (37-42 weeks of gestation, cephalic presentation, and no clinical or obstetric contraindications requiring cesarean section). Vaginal deliveries are highlighted for their health advantages for both mothers and newborns.^( 11 )^ Cesarean sections are recommended only when medically indicated to avoid risks to maternal or neonatal health.^( 12 )^ The guidelines stress that the choice of delivery method should be an informed, shared decision between the pregnant woman and the medical team, supported by the best scientific evidence, prenatal monitoring, and continuous labor assessment to determine necessary interventions.^( 11 , 12 )^

The SUS also implements programs and policies, such as Rede Cegonha, recently converted to Rede Alyne,^( 13 )^ aimed at improving maternal and neonatal care and reducing unnecessary cesarean sections. Rede Alyne allocated resources for the establishment, expansion, and renovation of Normal Birth Centers (CPNs - *Centros de Parto Normais* ) designed for low-risk vaginal births. These centers prioritize natural childbirth, empower obstetric nurses to work autonomously in low-risk situations, and enable women to actively participate in childbirth planning.^( 14 )^ However, the uptake of CPN accreditation remains low, with fewer than 55 centers nationwide, predominantly concentrated in the northeast.^( 15 )^

Despite these efforts, cesarean section rates have continued to increase over the past 15 years, revealing significant regional disparities. A similar pattern of rising cesarean section rates observed in Brazil has also been reported globally, particularly in low- and middle-income countries (LMICs).^( 16 , 17 )^ A study of 169 countries estimated that 29.7 million births occurred via cesarean section in 2015, nearly double the number in 2000. In Latin America and the Caribbean, the rate reached 44.3%, whereas it remained as low as 4.1% in West and Central Africa.^( 17 )^ Some Middle Eastern and North African countries, such as Egypt, have reported rates exceeding 57.3%.^( 18 )^ In Europe, cesarean rates varied between 16.0% and 55.9% from 2015 to 2019, with some countries reporting increases and others declines.^( 19 )^ In South Asian countries, such as Bangladesh, Nepal, and Pakistan, rates have also risen significantly over the years.^( 16 )^ However, substantial within-country variations exist, which are often linked to economic disparities and healthcare facility types (public *versus* private).^( 18 , 20 )^ The rise in cesarean section rates is driven by an increasing proportion of institutional births and a greater reliance on cesarean deliveries within these facilities.^( 17 )^ Socioeconomic inequalities contribute further, with higher rates observed among wealthier populations.^( 20 )^

Compared with previous national studies,^( 5 - 7 )^ our findings provide a more comprehensive temporal perspective on delivery modes within the SUS. Earlier reports indicated that cesarean rates in the public sector were elevated but remained below 50%, with regional variations showing higher rates in the southeast and lower rates in the North and Northeast. Our study confirmed these patterns, showing an overall national cesarean section rate of 44.6% in 2023 with regional disparities consistent with prior observations. Notably, the Northeast experienced a marked increase over the study period, aligning with previously reported trends but extending the temporal coverage through the post-COVID-19 years. Similarly, the gradual decline in vaginal deliveries reported previously was corroborated by our data, which demonstrated a 39% reduction between 2009 and 2023. These comparisons underscore that, while some trends were previously identified, our analysis captures more recent shifts and confirms the persistence of regional inequalities within the public healthcare system.

Several factors may contribute to these high rates, including a persistent focus on obstetrician-led care, limited integration of midwives and obstetric nurses into the care model, and inadequate prenatal preparation for labor and natural birth. Additionally, myths surrounding cesarean sections, such as their perceived superiority in avoiding pain and complications, remain prevalent despite a lack of scientific support.^( 6 , 11 , 12 )^ A notable peak in cesarean section mortality rates occurred in 2021 during the second year of the COVID-19 pandemic, potentially reflecting the strain on hospital resources.

The exceptionally low cesarean section rate observed in Roraima, which declined from 19.4% in 2009 to 6.8% in 2023, represents a marked deviation from national and regional trends. This unusually low rate may reflect a combination of factors, including limited access to surgical facilities, differences in healthcare infrastructure, and a potentially stronger reliance on midwifery- and primary-care-based delivery models. While such low rates might indicate the successful avoidance of unnecessary cesarean sections, they could also indicate restricted access to timely surgical interventions when medically indicated. Further investigation of local healthcare practices, resource availability, and maternal health outcomes in Roraima is warranted to clarify whether this pattern represents an optimal model for low-risk vaginal births or a gap in access to essential obstetric care.

The results of this study showed that, on average, cesarean sections required more financial resources and led to longer hospitalizations. This suggests a greater burden on hospital infrastructure and higher complexity of recovery, with implications for both SUS and parturients. In this context and considering the increasing cesarean section rates far above the World Health Organization recommendations, policymakers may consider promoting evidence-based strategies to optimize cesarean section use, ensuring that it is performed when medically necessary. Although cesarean sections had numerically higher mortality rates, the lack of statistical significance suggests that other factors ( *e.g* ., preexisting maternal conditions) might influence outcomes rather than the delivery mode itself. It is not clear from the SUS procedure classification which parameters were used to define a delivery (vaginal or cesarean) as low-risk rather than high-risk. It is possible that variations in comorbidities and risk factors within the low-risk classification for both delivery modes may have hindered the detection of statistically significant differences between the two groups.

The preference for cesarean sections is further reinforced by cultural factors and logistical advantages such as predictability in scheduling and shorter labor durations. This trend is more pronounced in private and supplementary healthcare services, where provider schedules and hospital logistics may favor cesarean deliveries without clear medical indications. These practices have influenced obstetricians’ and patients’ preferences in the SUS, further perpetuating cesarean section trends.^( 6 )^ Broader demographic shifts in Brazil should also be considered. Over the past two decades, the fertility rate has declined from 2.32 to 1.57 children per woman, whereas the annual number of births has decreased from 3.6 million in 2004 to 2.6 million in 2022. During this period, the average maternal age at childbirth rose from 25.3 to 27.7 years.^( 21 )^ These changes, coupled with regional socioeconomic and healthcare disparities, have contributed to the observed trends in delivery modes.

Regions with stronger hospital infrastructures, such as the Southeast and South, tend to have more specialized and medium- to high-complexity services, which may facilitate higher cesarean rates. Conversely, the North and Northeast regions have broader primary healthcare coverage, which enables enhanced prenatal monitoring and educational initiatives. Primary healthcare is important in educating pregnant women on the risks and benefits of different delivery methods and in reducing unnecessary cesarean rates.^( 22 , 23 )^ Despite this, cesarean section rates continue to rise in these regions, reflecting challenges such as insufficient hospital resources and logistical barriers.^( 24 )^

The financial aspects of the SUS further complicate this issue. The SUS financing values for both vaginal and cesarean deliveries remain low, are rarely updated, and lag behind inflation. For instance, the fixed SUS funding for vaginal deliveries has increased marginally in the last 15 years, from R$ 403.09 (US$ 123.65) to R$ 443.40 (US$ 136.01) in 2009, after which it has remained stagnant.^( 8 )^ According to the SUS policies, the fixed amount of financing per procedure should be agreed upon by three levels of management (federal, state, and municipal). However, this practice has not been widely implemented and depends on the availability of funds. For example, the State of São Paulo, through the *Tabela SUS Paulista Program* ,^( 25 )^ increased reimbursement for vaginal and cesarean deliveries by fivefold and fourfold, respectively. Despite higher funding for vaginal deliveries, the initiative has not been sufficient to reverse the increasing cesarean section rates in São Paulo.

Addressing these systemic issues requires a multi-pronged approach, including enhanced funding, infrastructure improvements, and better integration of multidisciplinary evidence-based care models. Expanding access to CPNs and strengthening primary healthcare initiatives can improve education and support for natural childbirth. Moreover, ensuring consistent monitoring and evaluation of cesarean section indications and fostering equitable care standards across regions are critical for promoting maternal and neonatal health within the SUS framework.

Among the limitations of this study, the data were obtained from an administrative database that was subject to coding errors.^( 26 )^ Procedures such as childbirth (both vaginal and cesarean) followed by tubal ligation were excluded due to a lack of specifications regarding birth risk. The data did not provide information on key outcomes or the parturients’ journey after hospitalization, such as complications during hospital stay or rehospitalization following delivery. Although other important information regarding parturients and neonates is available within DataSUS, it is fragmented across information systems. For example, the SIH provides information regarding hospital LOS, mortality, and SUS financing values by procedure, including deliveries; the SIM gathers information related to fetal and maternal deaths; and the live birth information system^( 27 )^comprises information such as APGAR, Robson scale, birth weight, and prenatal care. However, it is not possible to link these databases because of the absence of a unique anonymized patient identifier.^( 15 )^

## CONCLUSION

This study highlights a significant decline in low-risk vaginal deliveries and an increasing trend in cesarean sections within the SUS from 2009 to 2023, with notable regional disparities. Despite policies promoting vaginal births in low-risk pregnancies, cesarean section rates have continued to increase, reflecting cultural preferences, healthcare infrastructure, and financial constraints. These findings underscore the need for strengthened prenatal education, expanded access to Normal Birth Centers, and improved financial incentives to optimize delivery practices. Addressing these challenges is crucial to ensure equitable, evidence-based maternal healthcare within the framework of Brazil’s Public Health System.

Future studies should investigate the determinants of cesarean overuse, including provider incentives, patient preferences, and institutional factors. Quantitative analyses can assess the impact of maternal socioeconomic factors ( *e.g* ., education level and municipality of residence) and hospital characteristics ( *e.g* ., delivery volume and cesarean rates) on delivery modes and outcomes. Qualitative research exploring the perspectives of obstetricians and mothers on decision-making processes can provide deeper insights into the motivations behind delivery choices. Examining the disparities across socioeconomic and geographic contexts could inform policies promoting maternal health equity.

## SUPPLEMENTARY MATERIAL

Regional and demographic disparities in low-risk hospital deliveries in Brazil’s Public Health System

Marina Martins Siqueira, Gustavo Yano Callado, Lucas Hernandes Corrêa, Gabriely Rangel Pereira, Eduardo Félix Martins Santana

**DOI: 10.31744/einstein_journal/2026GS1911**

Table 1S Absolute number of vaginal and cesarean deliveries in the SUS (2009-2023)

**Table:** 

Brazil Year	Vaginal delivery		Cesarean delivery		p value*
	freq.	%	freq.	%	
2009	1,258,102	68	603,806	32	< 0.001
2010	1,190,275	66	621,270	34	
2011	1,141,508	64	637,558	36	
2012	1,068,181	63	638,695	37	
2013	1,053,751	62	650,813	38	
2014	1,052,904	61	662,433	39	
2015	1,096,703	63	649,596	37	
2016	1,035,371	62	626,630	38	
2017	1,031,378	61	654,101	39	
2018	1,024,679	60	682,141	40	
2019	997,646	59	695,999	41	
2020	914,722	57	679,453	43	
2021	885,027	57	665,913	43	
2022	814,311	56	642,887	44	
2023	771,880	55	622,411	45	
Total	15,336,438	61	9,733,706	39	

*Trend χ^2^ test.

Table 2S Characterization of Brazilian regions

**Table:** 

Region	Socioeconomic and demographic features	States (Nº of states and municipalities)
North	Inhabitants: 17,354,884 Populational density: 4.5 inhabitants/km ^2^ GDP per capita: 33,123 Household income per capita: 1,302	States: Acre (AC), Amapá (AP), Amazonas (AM), Pará (PA), Rondônia (RO), Roraima (RR), Tocantins (TO) Nº of states: 7 Nº of municipalities: 450
Northeast	Inhabitants: 54,658,515 Populational density: 36.1 inhabitants/km ^2^ GDP per capita: 25,401 Household income per capita: 1,146	States: Alagoas (AL), Bahia (BA), Ceará (CE), Maranhão (MA), Paraíba (PB), Pernambuco (PE), Piauí (PI), Rio Grande do Norte (RN), Sergipe (SE) Nº of states: 9 Nº of municipalities: 1,794
South	Inhabitants: 29,937,706 Populational density: 53.2 inhabitants/km ^2^ GDP per capita: 55,942 Household income per capita: 2,167	States: Paraná (PR), Rio Grande do Sul (RS), Santa Catarina (SC) Nº of states: 3 States Nº of municipalities: 1,191
Southeast	Inhabitants: 84,840,113 Populational density: 91.8 inhabitants/km ^2^ GDP per capita: 63,327 Household income per capita: 2,237	States: Espírito Santo (ES), Minas Gerais (MG), Rio de Janeiro (RJ), São Paulo (SP) Nº of states: 4 Nº of municipalities: 1,668
Midwest	Inhabitants: 16,289,538 Populational density: 16.3 inhabitants/km ^2^ GDP per capita: 65,651 Household income per capita: 2,202	States: Goiás (GO), Mato Grosso (MT), Mato Grosso do Sul (MS), Distrito Federal (DF) Nº of states: 4 Nº of municipalities: 467

Source: Developed by authors, with data from the 2022 Census of the Brazilian Institute of Geography and Statistics < https://www.ibge.gov.br/estatisticas/sociais/trabalho/22827-censo-demografico-2022.html >

Figure 1SMap of Brazilian states and regions
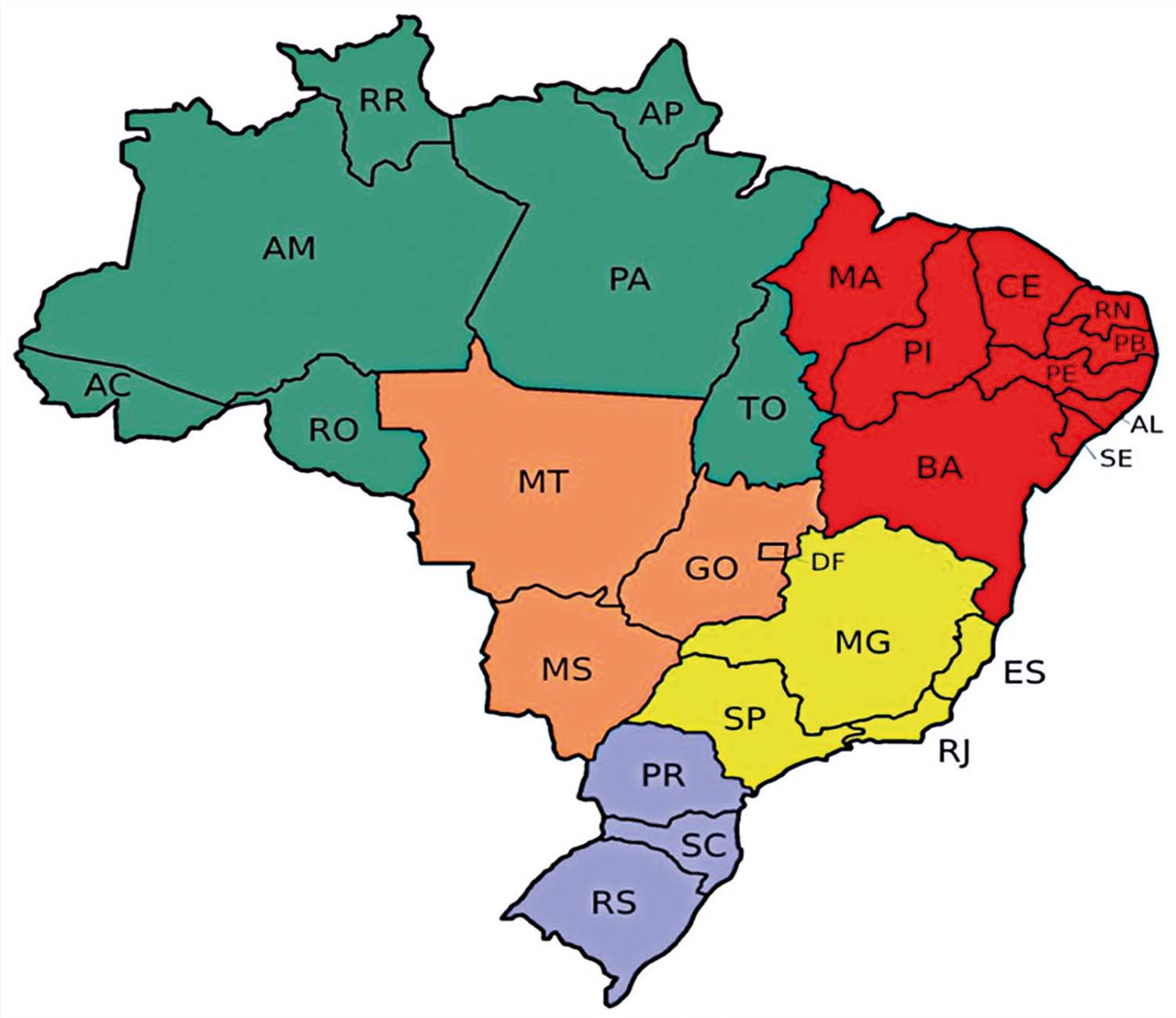
Source: https://www.infoescola.com/geografia/mapa-do-brasil/ Regions by color: northeast, red; north, green; midwest, orange; southeast, yellow; and south, purple.

Table 3S Average SUS financing value, length-of-stay, and in-hospital mortality rate by Brazilian region and mothers’ age group (2023)

**Table:** 

Average SUS financing value (2023)									
Delivery	Region	<15 years	15-19 years	20-24 years	25-29 years	30-34 years	35-39 years	40+ years	Total
Vaginal	North	$170.56	$172.51	$173.88	$174.13	$173.81	$174.64	$175.16	$173.62
	Northeast	$163.85	$163.92	$164.09	$164.27	$163.90	$164.22	$163.89	$164.08
	Southeast	$176.78	$176.03	$175.66	$175.18	$175.40	$175.42	$176.63	$175.56
	South	$180.01	$178.50	$178.96	$179.07	$178.79	$178.97	$179.71	$178.92
	Midwest	$172.51	$172.80	$173.82	$174.07	$174.24	$174.94	$176.25	$173.87
**Delivery**	**Region**	**<15**	**15-19**	**20-24**	**25-29**	**30-34**	**35-39**	**40+**	**Total**
C-section	North	$218.43	$224.07	$225.17	$226.54	$228.35	$232.11	$234.65	$226.51
	Northeast	$215.20	$216.98	$217.21	$217.44	$217.40	$218.36	$219.06	$217.43
	Southeast	$228.72	$227.19	$225.90	$226.29	$228.16	$230.81	$233.12	$227.34
	South	$232.00	$231.35	$229.23	$229.39	$230.47	$231.83	$234.16	$230.19
	Midwest	$215.74	$219.32	$220.31	$221.17	$221.96	$226.65	$228.14	$221.39
**Average LOS (2023)**									
**Delivery**	**Region**	**<15**	**15-19**	**20-24**	**25-29**	**30-34**	**35-39**	**40+**	**Total**
Vaginal	North	1.96	2.01	2.02	2.00	2.00	2.10	2.12	2.02
	Northeast	1.91	1.85	1.83	1.81	1.84	1.86	1.90	1.83
	Southeast	2.70	2.42	2.37	2.33	2.33	2.34	2.46	2.36
	South	2.41	2.20	2.16	2.12	2.12	2.15	2.17	2.15
	Midwest	2.15	2.07	2.04	2.01	1.99	2.04	2.09	2.03
**Delivery**	**Region**	**<15a**	**15-19a**	**20-24a**	**25-29a**	**30-34a**	**35-39a**	**40+a**	**Total**
C-section	North	2.63	2.74	2.71	2.69	2.71	2.89	3.00	2.73
	Northeast	2.51	2.53	2.51	2.48	2.50	2.59	2.67	2.52
	Southeast	2.77	2.65	2.60	2.59	2.60	2.72	2.81	2.63
	South	2.61	2.53	2.51	2.50	2.51	2.59	2.74	2.53
	Midwest	2.44	2.54	2.57	2.62	2.64	2.77	3.04	2.62
**In-hospital mortality rate (2023)**									
**Delivery**	**Region**	**<15a**	**15-19a**	**20-24a**	**25-29a**	**30-34a**	**35-39a**	**40+a**	**Total**
Vaginal	North	0.00	0.39	0.33	0.33	0.36	0.57	0.91	0.37
	Northeast	0.00	0.28	0.18	0.23	0.53	0.42	0.53	0.29
	Southeast	0.00	0.14	0.15	0.23	0.05	0.14	0.00	0.15
	South	0.00	0.22	0.10	0.04	0.13	0.00	0.00	0.10
	Midwest	0.00	0.26	0.10	0.33	0.22	0.23	0.00	0.21
**Delivery**	**Region**	**<15a**	**15-19a**	**20-24a**	**25-29a**	**30-34a**	**35-39a**	**40+a**	**Total**
C-section	North	0.00	0.33	0.56	0.24	0.70	1.20	0.49	0.50
	Northeast	0.00	0.21	0.26	0.35	0.51	0.25	0.33	0.32
	Southeast	1.31	0.30	0.21	0.26	0.45	0.78	0.15	0.34
	South	0.00	0.47	0.20	0.37	0.19	0.33	1.03	0.31
	Midwest	0.00	0.23	0.29	0.34	0.11	0.61	0.61	0.30

LOS: length-of-stay.

Table 4S Differences in SUS financing values (in US$) by delivery type across Brazilian regions (2023)

**Table:** 

Region	Total SUS financing value			Average SUS financing value	
	Vaginal delivery	C-section	Total	Vaginal delivery	C-section
North	$18,735,968.38	$18,924,515.45	$37,660,483.83	$173.62	$226.51
Northeast	$39,271,007.37	$42,443,621.94	$81,714,629.32	$164.08	$217.43
South	$16,641,958.05	$20,482,708.07	$37,124,666.13	$178.92	$230.19
Southeast	$47,398,143.37	$44,971,511.96	$92,369,655.33	$175.56	$227.34
Midwest	$10,714,973.82	$12,586,753.37	$23,301,727.18	$173.87	$221.39
Total	$ 132,762,050.99	$139,409,110.80	$272,171,161.79	$172.00	$223.98
p value*				[p= 0.008]	

* The average SUS financing value was statistically higher in the cesarean section than in the vaginal delivery group using the Mann-Whitney U test, with a significance level of p<0.05, using the currency conversion rate at the established date.

Table 5S Differences in mean length-of-stay by delivery type across Brazilian regions (2023)

**Table:** 

Region	Total LOS			Mean LOS	
	Vaginal delivery	C-section	Total	Vaginal delivery	C-section
North	217,548	228,062	445,610	2.02	2.73
Northeast	438,854	491,457	930,311	1.83	2.52
South	199,842	224,938	424,780	2.15	2.53
Southeast	637,173	519,331	1,156,504	2.36	2.63
Midwest	125,175	148,948	274,123	2.03	2.62
Total	1,618,592	1,612,736	3,231,328	2.10	2.59
p value*				[p=0.008]	

* Mean length-of-stay was statistically higher in the cesarean section group than in the vaginal delivery group using the Mann-Whitney U test, with a significance level of p<0.05.

LOS: length-of-stay.

Table 6S Differences in in-hospital maternal mortality by delivery type across Brazilian regions (2023)

**Table:** 

Region	Total deliveries			Vaginal deliveries			Cesarean deliveries		
	Procedures	Mortality		Procedures	Mortality		Procedures	Mortality	
	N	N	%	N	N	%	N	N	%
North	191,460	82	0.04	107,911	40	0.04	83,549	42	0.05
Northeast	434,553	132	0.03	239,347	69	0.03	195,206	63	0.03
South	181,997	37	0.02	93,015	9	0.01	88,982	28	0.03
Southeast	467,802	107	0.02	269,982	40	0.01	197,820	67	0.03
Midwest	118,479	30	0.03	61,625	13	0.02	56,854	17	0.03
Total	1,394,291	388	0.03	771,880	171	0.02	622,411	217	0.03
p value*				[p=0.180]					

* Relative mortality was similar in both groups according to the Mann-Whitney U test, with a significance level of p<0.05.
